# Plural dominance and the production of determiner-noun phrases in French

**DOI:** 10.1371/journal.pone.0200723

**Published:** 2018-07-30

**Authors:** Elisabeth Beyersmann, Britta Biedermann, F.-Xavier Alario, Niels O. Schiller, Solène Hameau, Antje Lorenz

**Affiliations:** 1 Department of Cognitive Science, Macquarie University, Sydney, Australia; 2 ARC Centre of Excellence in Cognition and its Disorders, Sydney, Australia; 3 School of Psychology and Speech Psychology, Curtin University, Perth, Australia; 4 Aix Marseille Université, CNRS, LPC, Marseille, France; 5 Leiden University Centre for Linguistics (LUCL), Faculty of Humanities & Leiden Institute for Brain and Cognition (LIBC), Leiden, The Netherlands; 6 Institut für Psychologie, Humboldt-Universität zu Berlin, Berlin, Germany; National Institutes of Health, UNITED STATES

## Abstract

In two experiments, we examined the functional locus of *plural dominance* in the French spoken word production system, where singulars and plurals share the same phonological word form. The materials included singular-dominant (singular more frequent than plural) and plural-dominant nouns (plural more frequent than singular). In Experiment 1, participants were instructed to produce determiner-noun phrases in response to singular and plural depictions of objects. In contrast to the dominance-by-number interaction that is typically observed in English, Dutch and German, the French picture-naming data revealed a main effect of number, but no effect of plural dominance. When participants were instructed to produce determiner-noun phrases in a reading aloud task (Experiment 2), where number is orthographically marked, a number-by-dominance interaction emerged. Our data suggest that plural dominance is encoded at the word form level within the context of recent theories of spoken word production.

## Introduction

The production of plural nouns, such as *tigers*, is usually delayed and more error-prone compared to the production of the corresponding singular form *tiger* [1,2]. One explanation for this number effect is that an explicit affixation process is needed when plural targets are produced (e.g., *tiger-s*). In the framework of the two-stage model of language production [3–5], this affixation process is assumed to occur at the word-form level of the mental lexicon, where stems and affixes are stored separately (see also [6,7]). In addition, plural forms are usually also more complex, and therefore possibly more difficult to retrieve at other levels of language production processing [1,8,9,10].

As demonstrated for different Indo-European languages, the number effect in language production is strongly modulated by the relative surface frequency of the singular and corresponding plural forms (e.g., *tiger* vs. *tigers*), an interaction referred to as "plural dominance" [1,2,11]. Usually, faster oral production of singulars compared to plurals is observed for singular-dominant nouns, where the singular is more frequent than the plural, but not for plural-dominant nouns (for similar data from a lexical decision task, see [12,13–15]). Comparable effects were reported in the naming error pattern of speakers with aphasia ([1,10,16,17]; for similar data from reading aloud, see [18]). This dominance of number is not merely a surface frequency effect because in spoken picture naming plural-dominant plural targets are not easier or faster to retrieve than their corresponding singular forms [1,17]. Instead, these studies concurrently suggest that plural formation is specifically challenged for singular-dominant nouns, whereas plural-dominant plural nouns are easier to retrieve and/or to generate, presumably due to representational differences in the mental lexicon.

**Fig 1:**
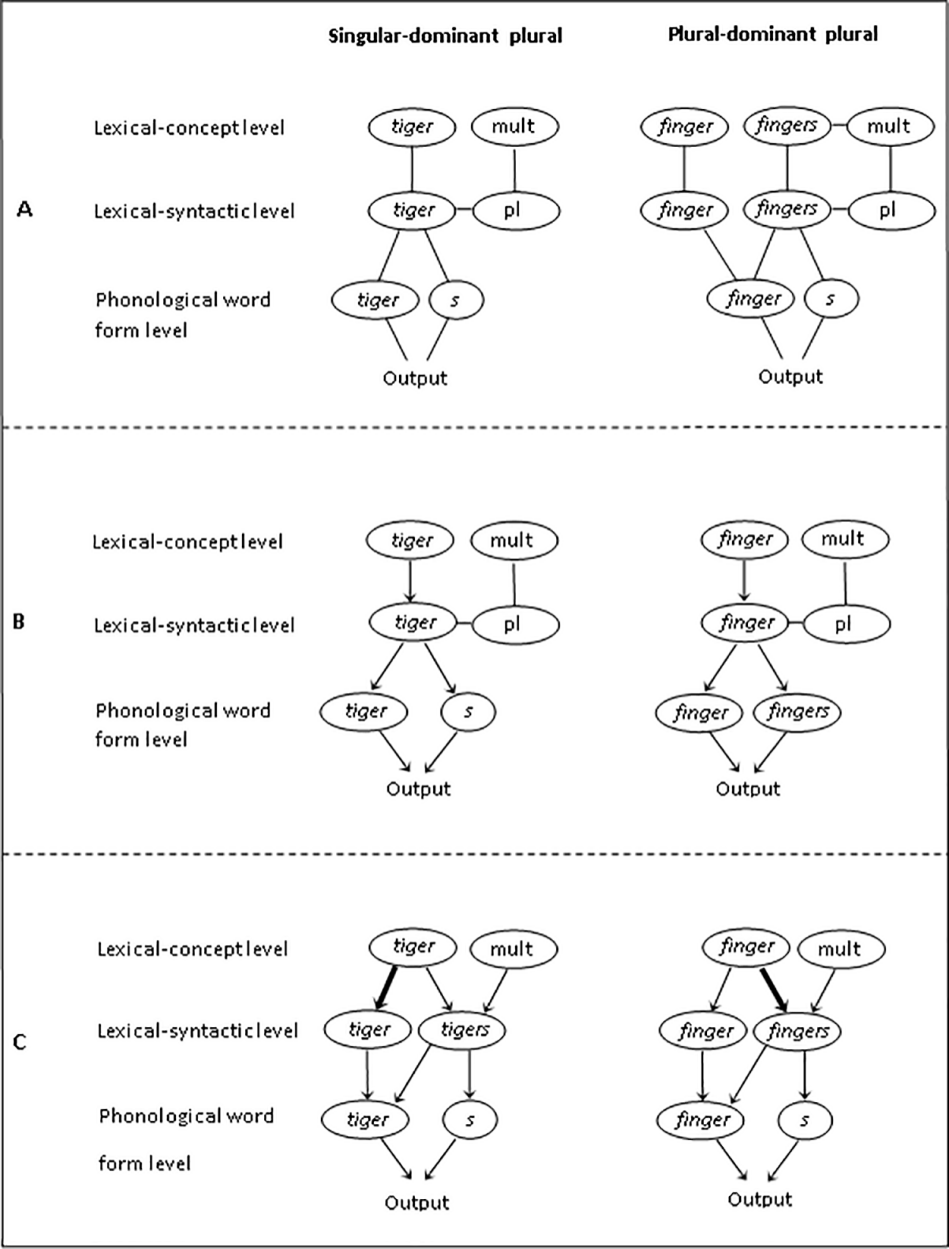
Morphological processing theories of spoken word production. Fig 1 is adapted from Beyersmann, Dutton, Amer, Schiller, and Biedermann [2]. Panel A represents the production of singular-dominant and plural-dominant plurals, based on a word production theory proposed by Levelt, Roelofs, and Meyer [4]. Panel B refers to a morphological processing theory of word production proposed by Biedermann, Beyersmann, Mason, & Nickels [1], and Panel C refers to a theory by Nickels, Biedermann, Fieder, and Schiller [9].

Outside of word reading, what continues to be a matter of debate however, is the underlying functional source of plural dominance effects in language production. Recent theories of morphological processing in spoken word production (for an overview, see Fig 1) accommodate two different representational nodes for regular plural forms at the word-form level (e.g., *tigers*): one node for the stem (*tiger*) and one for the plural suffix (-*s*) (for a related account, see [19])). Within the discrete two-stage model of language production [4], Biedermann et al. [1] suggested differences in the representation of singular- and plural-dominant nouns *at the word-form level*, with full listing for plural-dominant nouns and decomposition for singular-dominant plurals (see also [12])). A theoretical extension of this account is offered by Nickels, Biedermann, Fieder and Schiller [9], who proposed that dominance effects originate *in the links between concept and lemma level*. Nickels et al. proposed separate lemma representations for all singular and plural nouns regardless of dominance status (different to [4]), and that the obtained plural dominance effects in language production are assumed to result from differences in the activation strength of singular and plural lemmas as a function of plural dominance (see also [17])). The more frequently a word is produced, the stronger the links between concept and lemma level will be, resulting in faster and more accurate responses to high-frequency words. This account thus predicts stronger links between concepts and lemmas for singular-dominant singulars compared to their plurals, and for plural-dominant plurals compared to their singulars.

This study aims to shed light on the mechanisms underlying plural noun production in French with a focus on the locus of the plural dominance effect in the French word production system. Previous work was primarily based on data from English [1,9,16] and Dutch [2,8,11,12], and is therefore less suitable to account for the production of regular plurals in French. In French, the regular default plural form shares the phonological word form with the corresponding singular (e.g., *table*_sg_ [tabl] vs. *tables*_pl_ [tabl]). Therefore, the same processing costs are expected to arise for singulars and plurals at this level. Singular and plural nouns, however, differ at other levels of processing: just as in English and Dutch, they are expected to have different conceptual representations (single vs. multiple) and different lemma representations (singular vs. plural). In this respect, the French language affords unique conditions to test the differential predictions between the two theories, in contrast to the majority of Indo-European languages where regular plural morphemes are phonetically articulated (e.g., the plural suffixes in English, German or Dutch).

## Experiment 1: Picture naming

The goal of Experiment 1 was to examine the role of dominance in the production of French singular and plural nouns. Two different types of word pairs were compared: one condition in which the singular was more frequent than the plural (the singular-dominant condition) and one condition in which the plural was more frequent than the singular (the plural-dominant condition). While this paradigm has been previously used to study the role of plural dominance in language production in English, German, and Dutch, we are the first to use this paradigm within a group of French speakers (for data from comprehension, see [14])). French was chosen because, in contrast to languages such as English, German and Dutch, the French regular plural suffix is not phonologically marked, thus the spoken forms of singulars and plurals are indistinguishable. For this same reason, however, we needed to ensure that our French participants clearly differentiated between the phonologically identical singular and plural forms (e.g., *table*_sg_ [tabl] vs. *tables*_pl_ [tabl]). Thus, they were asked to produce the corresponding word determiner in addition to the target noun (*une table* [yn tabl] vs. *des tables* [de tabl]). In order to select the correct determiner (gender and number), participants had to retrieve the lexico-syntactic properties of the noun. Moreover, determiner selection in French is constrained by the phonological onset of the noun: for nouns beginning with a consonant the masculine determiner *un* is pronounced $\tilde{\varepsilon }$, whereas for nouns beginning with a vowel it is pronounced $\tilde{\varepsilon }n$. Effects of the phonological properties of the noun have been repeatedly observed in behavioural measures using determiner-noun phrases, suggesting that planning scope goes beyond the determiner in these kind of utterances [20,21]. Most critically, the basis for the direct comparison between the two types of words was the close matching of the two plural forms on surface frequency [16].

If it is true that plural-dominance originates at the lemma level [9], lexical selection of singular-dominant plurals should be more demanding at the lemma level due to weaker links between concepts and lemmas in the case of singular-dominant nouns. This account therefore predicts *longer* naming latencies for singular-dominant plural targets than for singular targets, but no number effect for plural-dominant nouns (i.e., a replication of the number-by-dominance interaction previously observed in English, German and Dutch). In contrast, if it is true that plural dominance originates at the word-form level [1], no difference would arise between singular-dominant and plural-dominant plurals, given the identical phonological word forms for singulars and plurals in French. In short, any observed differences between singulars and plurals in this task would imply that the relative frequency effect (i.e., dominance of number) is located at a higher conceptual or lexical level rather than at the phonological word form level.

### Method

This research was approved by the ethics committee of Aix-Marseille University.

#### Participants

Twenty-four students from Aix-Marseille University participated in this study. All participants had normal or corrected-to-normal vision and were native French speakers.

#### Materials

Target items were common nouns, selected from the LEXIQUE database [22]. Fifty-two plural-dominant and 52 singular-dominant words were selected. Each set consisted of 26 singular and 26 plural forms (see S1 Appendix). An argument raised by one of our reviewers is that optimal power would require a larger number of 1600 observations per cell [23] where we had only 624 observations per cell. The plural forms were composed of a stem (e.g., *canard* [Engl. *duck*]) and the regular plural-suffix–*s* (e.g., *canards* [Engl. *ducks*]). Plural-dominant plurals were more frequent than their singulars, *t*(47) = 3.80, and singular-dominant singulars were more frequent than their plurals, *t*(40) = 3.52, with a minimum difference of 0.14 logarithmic word frequency retrieved from LEXIQUE [22]. The mean item characteristics are presented in Table 1. A full list of materials is available at Figshare: https://figshare.com/s/c2ae0fe260286307cc4b.

**Table 1:** Mean item characteristics of French materials. Standard deviations are shown in parentheses.

*Property*	*Plural-dominant*	*Singular-dominant*
**Plural**		
Word frequency	34.66 (50.18)	36.17 (83.54)
Cumulative stem frequency	50.38 (69.00)	149.04 (245.87)
Phonological neighbourhood	6.46 (7.92)	7.85 (7.42)
Orthographic neighbourhood	2.12 (2.78)	3.15 (2.77)
Number of syllables	1.69 (0.68)	1.65 (0.63)
Number of phonemes	4.38 (1.39)	4.35 (1.20)
Number of letters	7.38 (1.72)	7.04 (1.28)
Age of acquisition	4.31 (1.69)	4.62 (1.60)
Visual complexity	2.12 (0.55)	2.21 (0.60)
Name agreement	0.96 (0.05)	0.97 (0.05)
**Singular**		
Word frequency	14.47 (20.13)	112.80 (171.43)
Cumulative stem frequency	50.38 (69.00)	149.04 (245.87)
Phonological neighbourhood	6.46 (7.92)	7.85 (7.42)
Orthographic neighbourhood	2.35 (3.70)	3.62 (3.38)
Number of syllables	1.69 (0.68)	1.65 (0.63)
Number of phonemes	4.38 (1.39)	4.35 (1.20)
Number of letters	6.38 (1.72)	6.04 (1.28)
Age of acquisition	4.31 (1.69)	4.62 (1.60)
Visual complexity	1.71 (0.53)	1.84 (0.61)
Name agreement	0.97 (0.03)	0.98 (0.04)

For each target, a picture was selected. Pictures were colour photographs representing either single or multiple exemplars. For all targets, we collected name agreement, visual complexity and age of acquisition ratings from 24 native French speakers (see Table 1). Visual complexity ratings were based on a 1–5 point scale, with increasing number indicating increased complexity. Age of acquisition ratings were based on a 1–7 point scale, with increasing number indicating increased acquisition age. Pictures were named with at least 80% accuracy.

The two lists of plurals (plural-dominant vs. singular-dominant) were matched on surface frequency. Moreover, plurals (plural-dominant vs. singular-dominant) and singulars (plural-dominant vs. singular-dominant) were matched on phonological neighbourhood, orthographic neighbourhood, syllable number, phoneme number, number of letters, name agreement, visual complexity and age of acquisition (see Table 1). To avoid stem repetition (i.e., singular and plural forms always shared the same stem), we created two counterbalanced lists.

#### Procedure

Before the experiment, informed written consent was obtained from all our participants. They were then asked to read through a picture book in which the pictures of 18 singular-plural paired items were presented together on a page. The goal of presenting the picture book was to ensure participants would be familiar with the task involving the production of singular and plural targets in response to singular and plural depictions of objects [8]. The items presented in the picture book were different from the experimental items. The singular was shown on the upper half, and the plural on the lower half of the page, with the corresponding indefinite singular or plural determiner noun phrase printed underneath each picture. Participants were instructed to look at each picture and read out aloud the corresponding determiner and noun.

*Stimuli were presented centrally on-screen using DMDX [24]. Each trial consisted of a 200 ms fixation cross, followed by a blank screen for 600 ms, followed by the target picture. Targets were presented in randomised order on a black background for a maximum of three seconds. Between trials, a blank screen was presented for 1,500 ms. Participants were instructed to name every picture as quickly and accurately as possible. Given that in French, singular and plural nouns are phonologically indistinguishable (the final plural–*s *in French in silent)*, *participants were asked to produce the corresponding word determiner in addition to the target noun (*un_*masc*_ *and* une_*fem*_ *for singular nouns*, *and* des_*masc/fem*_ *for plural nouns)*. *Responses were recorded with a head-worn microphone*. *The amplifier was configured individually for each participant*.

### Data analyses

Accuracy and reaction times (RTs) of vocal responses were corrected using CheckVocal [25]. Due to technical difficulties, the voice recordings from five participants were non-interpretable and therefore excluded. Incorrect responses (8.4%) were removed from reaction time analyses. Inverse RTs (1/RT) were calculated for each participant to correct for RT distribution skews and used throughout the analyses. Reaction times (RTs) and error rates (ER%) were analysed for each participant (Table 2).

**Table 2:** Spoken picture naming and reading aloud of French singular and plural nouns. Reaction times (RTs in ms) and error rates (ER in %), averaged across items for each participant. Standard deviations are presented in parentheses.

Number	singular-dominant		plural-dominant	
	RTs	ER%	RTs	ER%
picture naming				
Singular	994 (145)	5.3 (5.8)	936 (174)	7.7 (5.1)
Plural	1012 (120)	10.1 (9.3)	994 (158)	10.5 (11.2)
Difference	18	4.8	58	2.8
reading aloud				
Singular	669 (97)	2.1 (5.1)	699 (106)	2.4 (5.5)
Plural	683 (99)	2.8 (5.3)	671 (101)	2.1 (4.7)
Difference	14	0.7	-28	-0.3

Linear mixed-effect modelling was employed to perform the main analyses [26,27]. Fixed effects, random effects, and random slopes were only included if they significantly improved the model’s fit in a stepwise model selection procedure. Models were selected using chi-squared log-likelihood ratio tests with maximum likelihood parameter estimation. Following Barr et al. [28], we included the maximal random effect structure justified by the design. The model was refitted after excluding data-points whose standardised residuals were larger than 2.5 in absolute value [see 26], which led to the removal of 0.9% of the data. Linear mixed-effects models as implemented in the lme4 package [29] in the statistical software R (Version 3.0.3; [30]) were fitted using the above described selection procedure. Two fixed effect factors were examined: factor number (singular, plural) and factor dominance (singular-dominant, plural-dominant). The lmer default coding for treatment contrasts was used (i.e. reference ‘plural’ for factor number; reference ‘plural-dominant’ for factor item type). *P*-values were determined using the package lmerTest [31].

### Results

#### Reaction times

In the reaction time analyses, the inclusion of dominance, as well as the interaction between number and dominance did not significantly improve the model’s fit and thus these factors were excluded from the model. The results revealed a significant effect of number (*t* = 2.29, *p* = .036), showing that plurals were produced significantly more slowly than singulars. No other effects were significant.

**Fig 2:**
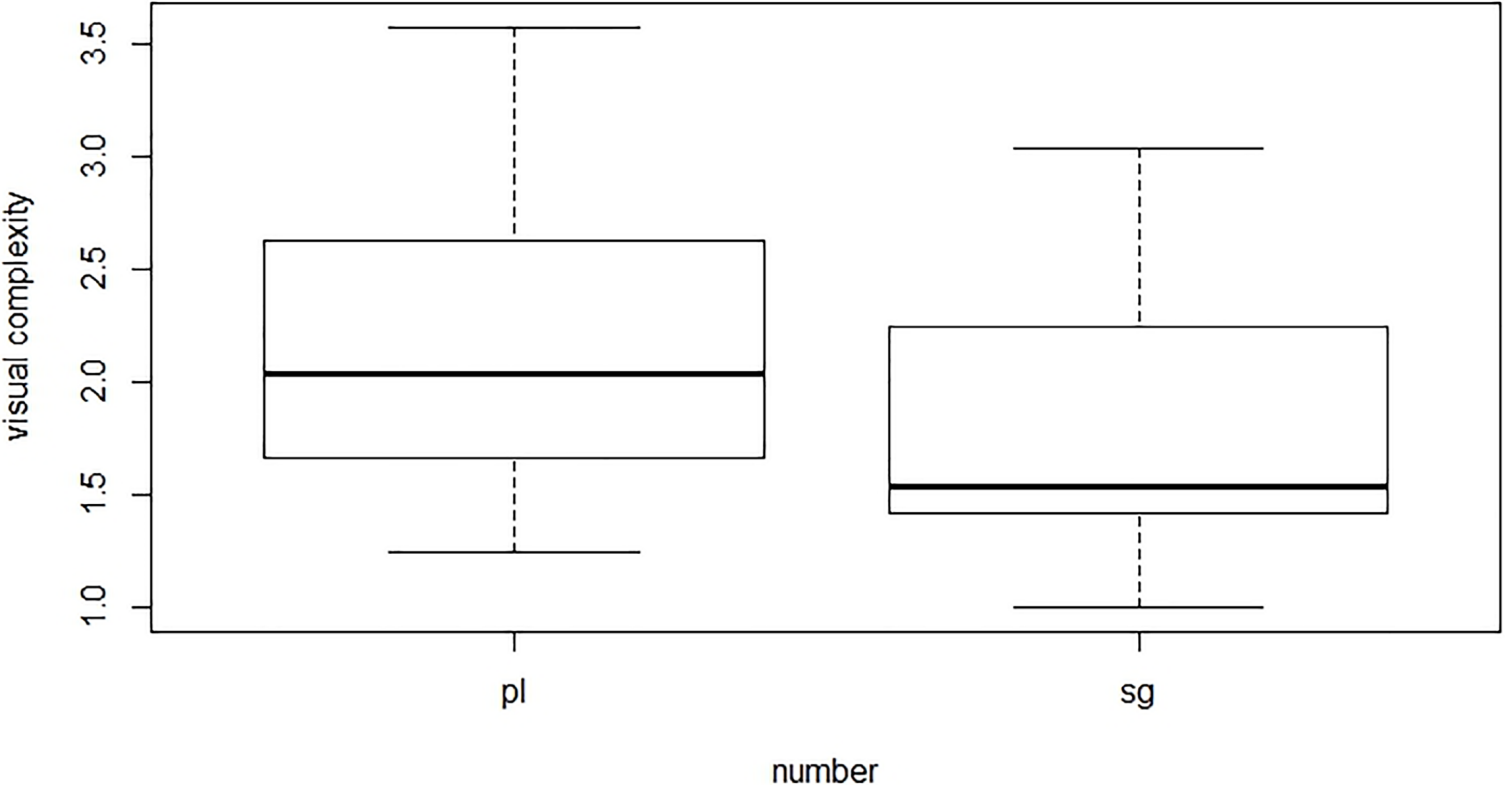
Distribution of visual complexity for plural and singular items in the picture naming task.

To further explore the nature of the number effect, we included visual complexity as a covariate in the analyses. As previously argued by Schiller and Caramazza [32,33], plural pictures are naturally higher in visual complexity than singular pictures (see Table 1). Visual complexity and number were weakly to moderately correlated (i.e. the point-biserial correlation was *r* = .313, *p* = .024). The distribution of visual complexity for singular and plural items is illustrated in Fig 2. The covariate analyses indeed revealed that the effect of visual complexity was significant (*t* = 5.32, *p* < .001). Importantly, the inclusion of factor visual complexity showed that the effect of number was no longer significant (*t* = 0.18, *p* = .859).

#### Error rates

The error analyses were performed based on the same principles as the RT analyses. We applied a binomial variance assumption to the trial-level binary data using the function glmer as part of the R-package lme4. First, we analysed the overall error rates in each condition. As in the reaction time analyses, the inclusion of dominance, as well as the interaction between number and dominance did not significantly improve the model’s fit and these factors were excluded from the model. In addition, the inclusion of the by-subject random slopes for factor number did not improve the model’s fit and were excluded. There was a significant effect of number (*z* = 2.04, *p* = .042), showing that participants made fewer errors responding to singulars than to plurals, which is consistent with the effect on response times. Once again, when factor visual complexity was included, a significant effect of visual complexity emerged (*z* = 3.12, *p* = .002) whereas the effect of number was no longer significant (*t* = 0.69, *p* = .491). No other effects were significant.

Second, we carried four additional analyses for the following four error types: no responses, word errors (e.g., *abeille* [engl. bee] instead of *mouche* [engl. fly]; cut responses such as *mou*… instead of *mouche*), determiner errors (e.g., *deux camions* [engl. two trucks] instead of *des camions* [engl. trucks]; *des jambes* [engl. legs] instead of *une jambe* [engl. a leg]), and fluency errors (e.g., *une mmmmme… méduse* [engl. a jjje… jellyfish]). The mean error rates for each error sub-type are presented in Table 3. Each type of error was then used as a dependant variable and the analyses were performed by applying a binomial variance assumption to the trial-level binary data, as above. Only the fluency error analysis revealed a significant main effect of number, showing that participants made more errors responding to plurals then to singulars (z = 2.35, p = .019). No other effects were significant.

**Table 3:** Error rates (in %) for each error type of Experiment 1, averaged across items for each participant. Standard deviations are presented in parentheses.

Number	singular-dominant				plural-dominant			
Type of error	no response	word error	det.* error	fluency error	no response	word error	det.* error	fluency error
Singular	2.02 (3.48)	1.62 (3.22)	0.81 (2.43)	0.81 (2.43)	1.62 (3.22)	1.62 (3.22)	2.43 (3.67)	2.02 (4.32)
Plural	1.62 (3.22)	3.64 (5.36)	1.21 (2.88)	3.64 (9.02)	2.43 (5.16)	2.02 (5.64)	2.43 (3.67)	4.05 (6.96)
Difference	0.40	-2.02	-0.40	-2.83	-0.81	-0.40	0.00	-2.03

*det. = determiner

### Discussion

The central finding of Experiment 1 is the main effect of number, showing that on average plurals were responded to more slowly than singulars. This effect cannot originate from the word-form level because the French word forms for plural and singular are identical. Furthermore, it is also not likely that the effect originates from specific difficulties in retrieving the plural determiner compared to singular determiners. In fact, determiner selection would predict the exact opposite of the number effect observed in Experiment 1 because determiner retrieval would be more demanding for singular compared to plural targets. For singular targets, two different singular determiner forms are available for selection (*un* and *une*; LEXIQUE logarithmic lexical frequencies: 9.5 and 9.2), whereas for plural targets, only one determiner form for both gender classes exists (*des*; LEXIQUE logarithmic lexical frequency = 9.3) (for more detailed considerations of these issues, see [21,34]).

Most crucially, the results of Experiment 1 demonstrate that the typical number-by-dominance interaction is *not* observed in a spoken picture naming task with French noun plurals. Our findings therefore differ from previous results from English, Dutch and German, where a robust number-by-dominance interaction has been repeatedly observed [1,2,16,17]. The obvious difference between French and other previously investigated languages is that the spoken word forms of plurals and singulars in French are identical. The word form level theory [1] suggests differences between singulars and plurals would be expected to emerge in languages with distinct singular and plural word forms, but not in languages with identical singular and plural word forms, and can therefore account for the present findings. In addition, we observed a main effect of visual complexity (for a similar result from picture naming in German and Dutch, see [32,33]), suggesting that the longer response times for plurals were due to differences in visual and/or semantic complexity of plural and singular targets [8].

Given the absence of an effect of plural dominance in Experiment 1, we designed a second experiment to further examine the hypothesis that plural dominance may originate from word form processing. Although in French the spoken word forms of singular and plurals do not differ, the orthographic word forms of singulars and plurals are clearly distinct (e.g., *table* vs. *tables*), in fact very often just as distinct as they are in English. We therefore designed a reading aloud experiment using the same materials as in Experiment 1, in which participants were exposed to the orthographic form of the target words and then asked to read the printed words out aloud. If it is indeed true that the dominance effect is located at the word form level, we would expect to obtain the typical number-by-dominance interaction in reading aloud due to differences of the orthographic word forms (see also [14])).

## Experiment 2: Reading aloud

### Method

This research was approved by the ethics committee of Aix-Marseille University.

#### Participants

Thirty-six students from Aix-Marseille University participated in this study. All participants had normal or corrected-to-normal vision and were native French speakers.

#### Materials

The same 52 singular-plural pairs as in Experiment 1 were used (see S1 Appendix), but presented as printed word forms instead of pictures. To discourage participants from reading out the presented words based on sub-lexical grapheme-to-phoneme correspondences, we included a second set of 52 pairs of singular-plural fillers with irregular pronunciations (e.g. *cerf*, *ciel*, *clef*, etc.). Following Lupker et al. [35], who suggest that the inclusion of irregular words invokes a "lexical-checking" strategy, words with irregular pronunciations were chosen such that the MANULEX grapheme-to-phoneme consistencies for each word [36] were below 80. The average MANULEX grapheme-to-phoneme consistency for the words used in Experiment 1 was 85.5 (SD: 9.9), but only 60.4 (SD: 11.1) for the additional irregular fillers. Regular and irregular plurals, as well as regular and irregular singulars, were matched on number of letters, number of phonemes, number of syllables, cumulative stem frequency, surface frequency, orthographic neighbourhood and phonological neighbourhood.

#### Procedure

Stimuli were presented centrally on-screen using DMDX [24]. Printed words were presented without determiners for reading aloud and participants were asked to produce determiner-noun phrases. Thus, they had to retrieve grammatical gender and the corresponding singular or plural determiner form in addition to the target noun.

Targets were presented in randomised order on a black background until the first response or for a maximum of three seconds. Participants were instructed to respond as quickly and accurately as possible. Responses were recorded with a head-worn microphone. The amplifier was configured individually for each participant. To avoid that participants would be exposed to the same stem twice, we created two counterbalanced lists.

#### Data analyses

The analyses of Experiment 2 were conducted following the same procedures as Experiment 1. Accuracy and response times of vocal responses were corrected using CheckVocal [25]. Irregular fillers and incorrect responses (2.4%) were removed from reaction time analyses. Inverse RTs (1/RT) were calculated for each participant to correct for RT distribution skews and used throughout the analyses. Data points whose standardised residuals were larger than 2.5 in absolute value were excluded which led to the removal of 2.0% of the data. Reaction times (RTs) and error rates (ER%) were analysed for each participant (Table 2).

### Results

In the reaction time analyses, the inclusion of by-subject random slopes for factors dominance and number as well as their interaction did not significantly improve the model’s fit and were therefore excluded. The analyses revealed a significant interaction between dominance and number (*X*^2^(1) = 18.37, *p* < .001), showing that in the singular-dominant condition, singulars were produced faster than plurals (*t* = 2.12, *p* = .034), whereas in the plural-dominant condition, plurals were produced faster than singulars (*t* = 3.94, *p* < .001). No other effects were significant. In the error data, there were no significant effects (all *z* < 1).

#### Cross-experiment analysis

To more directly compare the contrasting pattern of findings in Experiments 1 and 2, we carried out a post-hoc analysis across both data sets. A linear mixed-effects model was created with three fixed effect factors (number: singular, plural; dominance: singular-dominant, plural-dominant; task modality: picture naming, reading aloud), their interactions, random slopes, and random intercepts for participants and items. The lmer default coding for treatment contrasts was used (i.e. reference ‘plural’ for factor number; reference ‘plural-dominant’ for factor item type; reference ‘picture naming’ for factor task modality). In the reaction time analyses, the inclusion of by-subject random slopes for factors number, dominance, task modality and their interactions did not significantly improve the model’s fit and were excluded. The analyses revealed that there was a significant three-way interaction between number, item type and task modality (*X*^2^(1) = 5.48, *p* = .019), showing that the absence of interaction between number and item type in Experiment 1 (picture naming; *t* = 0.03, *p* = .974) contrasted with the significant interaction seen in Experiment 2 (word reading; *t* = 4.12, *p* < .001). There was a significant interaction between task modality and number (*X*^2^(1) = 19.56, *p* < .001) and a significant main effect of number (*X*^2^(1) = 6.98, *p* < .001). There was also a significant main effect of task modality (*X*^2^(1) = 59.11, *p* < .001). No other effects were significant.

In the error data, there was a significant main effect of task modality (*X*^2^(1) = 19.25, *p* < .001), showing that participants made more errors in the picture naming task than in the reading aloud task. No other effects were significant.

The key result is the significant three-way interaction between number, item type and task modality. While there was no significant interaction between number and item type in Experiment 1 (picture naming), this interaction was indeed significant in Experiment 2 (reading aloud). The singular-advantage in the plural-dominant condition of Experiment 1 was reversed in Experiment 2 (see Table 2).

## General discussion

The goal of our study was to clarify the functional locus of the plural dominance effect within the spoken word production system, with the purpose of understanding the representation of suffix morphology in the language production system. The plural dominance effect was newly tested using a language with identical phonological word forms for singular and plurals, using a spoken picture naming task (Experiment 1) and a word reading task (Experiment 2). The results revealed a significant three-way interaction between number (singular, plural), item type (singular-dominant, plural-dominant) and task modality (picture naming, word reading), showing that the absence of an interaction between number and item type in the picture naming task contrasted with the presence of a significant interaction between number and item type in word reading.

Given that French singular and plural forms are phonologically identical, such results allow for a clear-cut discrimination between the lemma level theory by Nickels et al. [9] and the word form level theory by Biedermann et al. [1]. The lemma level theory assumes that the obtained plural dominance effects in language production results from differences in the activation strength of singular and plural lemmas as a function of plural dominance (see also [17])). This theory predicts that an effect of plural dominance should also arise in French picture naming, which is clearly inconsistent with the results of Experiment 1. In contrast, the word form level theory [1] suggests that the dominance effect is located at the word form level, such that differences between singulars and plurals would be expected to emerge in languages with distinct singular and plural word forms, but not in languages with identical singular and plural word forms, and can therefore account for the results of Experiment 1. This is also in line with the results of Experiment 2, showing that in the singular-dominant condition, singulars were produced faster than plurals, whereas in the plural-dominant condition, plurals were produced faster than singulars (see Table 2). These data thus confirm the hypothesis that in French reading aloud the dominance effect originates at the orthographic word form level.

Our French data provide evidence for a clear singular-advantage for singular-dominant words, which is consistent with what has been previously observed in English, Dutch and German picture naming studies [1,2,10,17]. However, the plural-advantage seen in the French plural-dominant condition differs from the typically equivalent response times between plural-dominant singular and plural in English, Dutch and German. One possible explanation for the diverging results is the different response formats. Our French participants were asked to produce determiner-noun phrases, whereas English, Dutch and German participants were asked to produce nouns in isolation, precisely because the phonological forms of singular and plural words in isolation are identical. Singular and plural determiners were balanced across dominance types and can therefore not account for the interaction observed in Experiment 2. However, as previously argued by Schiller and Caramazza [33] in the context of noun phrase production in German and Dutch, the production of determiner-noun phrases in French can provide an overall boost in the production of plurals compared to singulars because plural-determiner selection (only one plural determiner form: *des*) is more straightforward than singular-determiner selection (choice between two gender-marked singular determiner forms: *un* and *une*) (for further discussion, see [34])). Such boost could modify the shape of the interaction between dominance and number. Indeed, related results from a French lexical decision task by New et al. [13] demonstrate that, when participants are asked to make a visual lexical decision on target nouns without determiners, the typical dominance pattern is observed. New et al. report a significant singular-advantage in the singular-dominant condition, but no difference between the singulars and plurals in the plural-dominant condition, which is exactly consistent with the pattern previously reported in English, Dutch and German picture naming. Hence, the use of determiners in our study may explain why a plural advantage emerged in the plural-dominant condition in Experiment 2, thus changing the shape of the interaction between dominance and number in these data. The shape of interaction notwithstanding, the key observation in our present study is that the observed dominance effect originates at the orthographic word form level of the French word production system.

One limitation of our study is that although our findings shed light on the representation of number in French, it is difficult to use these data to make predictions about the structure and processing mechanisms of the plural production system in other languages. First, in contrast to languages such as English, German, and Dutch, the spoken forms of French singulars and plurals are impossible to distinguish, because the plural suffix is not phonologically marked. Therefore, the representation of number in the French language production system may be fundamentally different from the representation of number in languages in which the plural suffix is phonologically marked. While our results suggest that the observed dominance effect in reading aloud originates at the orthographic word form level in French, they do not necessarily infer that similar principles apply to the production of plurals in other languages. Second, in contrast to previous experiments from English, German, and Dutch, where the role of plural dominance has been examined using bare noun production tasks, our present experiments involved the production of determiner-noun phrases. While a no determiner variant of our French experiment would have been desirable, this was not possible because in French the singular and plural forms are indistinguishable. That is, not only it would have been impossible to distinguish correct vs. incorrect responses, but also participants would have been able to develop a response strategy to perform the task without drawing on their morphological knowledge. Most importantly however, although these differences in task demands make it difficult to generalise our findings to previous results from other languages, it is unlikely that the plural dominance effect in Experiment 1 was washed out by the inclusion of determiners in the task, because as we argued above the production of determiner-noun phrases can only provide an overall boost in the production of plurals compared to singulars [33], which were balanced across dominance types. Moreover, a widely accepted assumption within models of plural production typically is that lemmas have uni-directional pointers that can activate optional determiner nodes, indicating that, from the perspective of the model tested, the requirement to produce a determiner does not modulate noun retrieval (e.g., [4]; for a detailed illustration see Fig 9 in [9]).

## Summary and conclusions

The results of our present study suggest that obvious differences exist between the French plural production system and the plural production system of other Indo-European languages including German, English and Dutch. In contrast to German, English and Dutch, the French phonological forms of singular and plural nouns are identical. Hence, a straightforward explanation for the presence of plural dominance effects in German, English and Dutch picture naming (as revealed in previous work), as well as the absence of plural dominance effects in French picture naming (as revealed in our present work), is that, at least in French, plural dominance is encoded at the word form level within the spoken word production system. This conclusion is substantiated by the fact that when the form does explicitly encode plural (i.e. orthographic form) then the interaction is observed.

## Supporting information

S1 Appendix This file contains a supplementary table. (DOCX)
