# Supplementary material for: Plural dominance and the production of determiner-noun phrases in French
Source: PLoS One. 2018 Jul 30;13(7):e0200723. doi: 10.1371/journal.pone.0200723 (PMC6066208; doi:10.1371/journal.pone.0200723)
Supplement: S1 Appendix — (DOCX) [file pone.0200723.s001.docx]

# **S1 Appendix**

|  | **Singular-dominant** | | **Plural-dominant** | |
| --- | --- | --- | --- | --- |
| **item** | **singular** | **plural** | **singular** | **plural** |
| 1 | un avion | des avions | un artichaut | des artichauts |
| 2 | un banc | des bancs | une carotte | des carottes |
| 3 | une boîte | des boîtes | une cerise | des cerises |
| 4 | un camion | des camions | un champignon | des champignons |
| 5 | un canard | des canards | une chaussette | des chaussettes |
| 6 | une chaise | des chaises | une chaussure | des chaussures |
| 7 | une chemise | des chemises | un clou | des clous |
| 8 | un cigare | des cigares | un doigt | des doigts |
| 9 | un coeur | des coeurs | un escargot | des escargots |
| 10 | une corde | des cordes | une étoile | des étoiles |
| 11 | une couronne | des couronnes | une feuille | des feuilles |
| 12 | une cravate | des cravates | une fleur | des fleurs |
| 13 | une église | des églises | une fourmi | des fourmis |
| 14 | une enveloppe | des enveloppes | une fraise | des fraises |
| 15 | une flèche | des flèches | un gant | des gants |
| 16 | une lampe | des lampes | une jambe | des jambes |
| 17 | une main | des mains | une méduse | des méduses |
| 18 | une montagne | des montagnes | une mouche | des mouches |
| 19 | un panier | des paniers | un nuage | des nuages |
| 20 | un pantalon | des pantalons | un oignon | des oignons |
| 21 | un serpent | des serpents | un palmier | des palmiers |
| 22 | un singe | des singes | un piano | des pianos |
| 23 | une table | des tables | un poulpe | des poulpes |
| 24 | un train | des trains | un requin | des requins |
| 25 | une valise | des valises | une roue | des roues |
| 26 | une voiture | des voitures | un scorpion | des scorpions |
